# SARS-CoV-2-induced SIADH: a novel cause of hyponatremia

**DOI:** 10.1007/s00391-021-01863-1

**Published:** 2021-03-01

**Authors:** Johannes Kleybolte, Benjamin Storek, Björn Hegner

**Affiliations:** 1 Vivantes Ida Wolff Hospital for Geriatric Medicine, Juchaczweg 21, 12351 Berlin, Germany; 2 Clinic for Nephrology and Intensive Care Medicine, Charité-Universitätsmedizin Berlin, corporate member of Freie Universität Berlin, Humboldt-Universität zu Berlin, and Berlin Institute of Health, Berlin, Germany

## Case history

An 86-year-old male admitted to the emergency department after stumbling and falling at home was diagnosed with fractures of the body of the humerus, dens axis and the posterior arch of the atlas. After osteosynthesis and immobilization with a cervical orthosis, the patient was transferred to the geriatric hospital for rehabilitation. He received tramadol (150 mg daily) for analgesia. Decompensated right heart failure was successfully treated with loop diuretics. His medical history included atrial fibrillation necessitating anticoagulation with rivaroxaban, type 2 diabetes mellitus treated with sitagliptin and hypothyroidism substituted with levothyroxine. Routine testing of pharyngeal swabs with the polymerase chain reaction (PCR) for severe acute respiratory syndrome coronavirus 2 (SARS-CoV-2) on admission and before discharge 7 days later, was negative.

The patient returned to the emergency department 2 days after discharge, presenting with exacerbated pain due to gonarthrosis and was readmitted to hospital. Tramadol was increased from 150 mg to 200 mg per day and physical therapy was started, resulting in sufficient pain relief. The complete medication on admission is listed in Table 1. Surprisingly, a pharyngeal swab now tested positive for SARS-CoV‑2. The patient was transferred to a quarantine unit but never developed symptoms of coronavirus disease 2019 (COVID-19), such as fever, cough, shortness of breath, nasal congestion, fatigue, diarrhea or smell and taste impairment.

**Table 1:** Medication on second admission

Drug	Dose	Morning—noon—evening
Rivaroxaban	15 mg	1—0—0
Sitagliptin	50 mg	1—0—0
L‑thyroxine	175 µg	1—0—0
Pantoprazole	40 mg	1—0—0
Torasemide	5 mg	1—1—0
Tramadol	50 mg	1—0—2

## Diagnostic findings

Blood pressure was 110/70 mm Hg, heart rate 56 beats per minute, respiratory rate 14 per minute, oxygen saturation 96% on ambient air, and body temperature 35.9 °C. Lungs, heart, and abdominal examinations were unremarkable. There were no signs of volume overload or depletion. Laboratory tests revealed elevated C‑reactive protein (CRP) with procalcitonin and white cell count within normal ranges (Table 2). Normocytic anemia was ascribed to surgery for the fracture 2 weeks earlier. The CRP spontaneously decreased without antibiotic treatment but lymphocytopenia and eosinopenia developed (Table 2), while interleukin‑6 (IL-6), D‑dimer, troponin‑T, NT-proBNP (N-terminal prohormone of brain natriuretic peptide), and lactate dehydrogenase (LDH) were elevated (Table 2), features frequently observed in patients with COVID-19 [9]. Progressive hypo-osmolar hyponatremia with a nadir of 128 mmol/L on day 13 (Tables 2 and 3) was noted. Diagnostic work-up revealed inappropriately high urine osmolality and natriuresis (Tables 2 and 3). Fractional excretion of uric acid (FEUA) was increased to 14.3%. Cortisol was slightly increased, ruling out adrenal insufficiency. Thyroid function tests showed slightly elevated thyroid-stimulating hormone (TSH) and free thyroxine (fT4) with reduced free triiodothyronine (fT3) suggesting underconversion of T4 to T3 during levothyroxine substitution. The patient reported constant moderate fluid intake over the last months.

**Table 2:** Laboratory findings

	Reference range	Unit	Day 1	Day 3	Day 5	Day 14
*Blood count*						
White blood cell count	3.90–10.50	/nL	6.7	5.40	2.91	4.46
Lymphocyte count	1.10–4.40	/nL	–	–	0.41	0.34
Eosinophile count	0.02–0.50	/nL	–	–	0.14	0.00
Hemoglobin	12.5–17.2	g/dL	8.4	8.2	9.0	10.3
Platelet count	150–370	/nL	339	342	333	224
D‑dimer	< 0.50	mg/L	–	–	2.44	–
*Clinical chemistry*						
Sodium	136–145	mmol/L	129	133	130	128
Potassium	3.5–4.5	mmol/L	3.1	3.5	3.6	3.8
Osmolality	280–300	mosm/kg	–	–	–	273
Creatinine	0.70–1.20	mg/dL	1.22	1.19	1.07	1.15
Troponin T	< 14	ng/L	–	–	108	–
NT-proBNP	< 526	ng/L	–	–	2861	–
LDH	135–250	U/L	–	–	289	–
Procalcitonin	< 0.50	µg/L	–	0.26	0.1	–
IL‑6	< 7.0	ng/L	–	–	17.3	–
CRP	< 5.0	mg/L	179.4	154.2	44.0	24.1
*Endocrinology*						
TSH	0.27–4.20	mU/L	–	–	–	6.05
fT3	2.00–4.00	ng/L	–	–	–	1.51
fT4	9.30–17.00	ng/L	–	–	–	20.20
Cortisol	–	nmol/L	–	–	–	710.0
Aldosterone	–	ng/L	–	–	–	37.0
Renin	–	ng/L	–	–	–	10.3
*Urine diagnostics*						
Osmolality	50–1400	mosmol/L	–	–	–	573
Sodium	71–171	mmol/L	–	–	–	122
Potassium	32–83	mmol/L	–	–	–	41
Uric acid	0.37–0.92	g/L	–	–	–	0.38
Creatinine	39.0–259.0	mg/dL	–	–	–	75.1
*Virology*						
SARS-CoV‑2 PCR	Negative	–	Positive	–	–	Positive

*NT-proBNP* N-terminal prohormone of brain natriuretic peptide, *LDH* lactate dehydrogenase, *IL-6* interleukin-6, *CRP* C-reactive protein, *TSH* thyroid-stimulating hormone, *fT3* free triiodthyronine, *fT4* free thyroxine, *SARS-CoV-2* severe acute respiratory distress syndrome coronavirus 2, *PCR* polymerase chain reaction

**Table 3:** Plasma sodium levels

	Reference range	Unit											
Day of hospital stay			−26	−8	1	3	5	8	13	14	15	19	21
Plasma sodium	136–145	Mmol/L	142	136	129	133	130	131	128	128	128	130	136

## Diagnosis

The findings were consistent with the syndrome of inappropriate antidiuretic hormone secretion (SIADH).

## Therapeutic interventions and course

Tramadol, a potential cause for the SIADH due to its central morphine receptor agonism and increase of serotonin release, was discontinued and metamizole was started at 1 g 4 times daily. Fluid intake was restricted to 1.2 L per day.

Plasma sodium concentrations slowly normalized over the following 8 days. The patient remained asymptomatic with respect to both hyponatremia and COVID-19. The PCR testing for SARS-CoV‑2 in pharyngeal swabs was first negative on day 33 and the patient was discharged home.

## Discussion

Clinical presentation of patients with COVID-19 ranges from asymptomatic to severe symptoms or death [9]. In addition to respiratory tract infections and systemic inflammation, multiple other organ systems, including kidneys, heart, vasculature, and the central nervous system (CNS), may be susceptible to SARS-CoV‑2 [5]. The SIADH has been described in patients with symptomatic [8] as well as asymptomatic SARS-CoV‑2 infection [4]. Geriatric patients are at the highest risk as age and age-related comorbidities are associated with hospitalization and death [6]. Hyponatremia is the most frequent electrolyte disturbance in clinical practice and is particularly relevant in the older population [7].

Before the SARS-CoV‑2 pandemic, the most likely causes of hyponatremia in the patient presented here would have been decompensated heart failure, dehydration caused by excessive diuretic treatment, hypothyroidism, and SIADH due to traumatic brain injury or tramadol. With the development of hyponatremia, the patient did not show volume overload or depletion although NT-proBNP was moderately elevated. Similar to troponin T, NT-proBNP was probably increased due to the SARS-CoV‑2 infection without clinically apparent signs of heart failure. Urinary sodium concentration and FEUA were both elevated militating against cardiac decompensation or hypotonic dehydration. There was evidence of impaired conversion of T4 to T3 potentially indicating a SARS-CoV‑2 associated non-thyroidal illness syndrome; however, TSH was only slightly elevated making clinically significant hypothyroidism unlikely. Instead, clinical and laboratory findings were suggestive of SIADH. A potential cause would have been brain injury incurred during the fall that resulted in fractures of the atlas and axis; however, computed tomography of the brain did not show any parenchymal lesions or intracranial hemorrhage and hyponatremia did not develop before day 18 following the trauma. Tramadol may have been the causative agent since it was started 10 days before onset of hyponatremia and sodium levels further declined after increase of the tramadol dose.

This case provides a rare opportunity to precisely document the temporal coincidence of SIADH and SARS-CoV‑2 infection suggesting a potential causal relationship. Both hyponatremia and SARS-CoV‑2 infection demonstrably occurred within a timeframe of 8 days. Although tramadol cannot completely be ruled out as the cause of SIADH, the chronology more convincingly argues in favor of SARS-CoV‑2 infection.

As potential mechanisms, inappropriate secretion of ADH resulting from left atrial underfilling subsequent to pulmonary vasoconstriction induced by lung injury as well as direct nonosmotic ADH release triggered by inflammatory cytokines such as IL‑6 have been discussed [8]. The report complements other cases of SIADH in COVID-19 [4, 8] since we documented elevated IL‑6 levels for the first time as a possible pathophysiologic link between SARS-CoV‑2 infection and SIADH. In a cohort of 29 COVID-19 patients, IL‑6 levels were inversely correlated with serum sodium concentrations and blockade of IL‑6 with tocilizumab was associated with an increase in sodium [2], further substantiating causality; however, whether or not hyponatremia was caused by SIADH had not been studied. It is conceivable that infection of the CNS with SARS-CoV‑2 can cause SIAHD. Impaired taste and smell, ataxia, dizziness, and loss of consciousness have been attributed to a probable neuroinvasive potential of SARS-CoV‑2 [1] and other viruses have been implicated in encephalitis with SIADH [3].

Diagnostic work-up of hyponatremia in a geriatric patient with multiple comorbidities and polypharmacy is a common clinical problem. Our case illustrates that SARS-CoV‑2 infection adds to the complexity of differential diagnoses and should also be considered.
